# Pubic symphysitis in the post-urological surgery setting: A case report and literature review

**DOI:** 10.1016/j.radcr.2025.08.062

**Published:** 2025-09-19

**Authors:** Asmae Guennouni, Omar Ibelkouchen, Soukaina Bahha, Mohamed Fadil, Laila Jroundi, Omar El Aoufir

**Affiliations:** Emergency Radiology Unit, Ibn Sina University Hospital Center, Rabat, Morocco

**Keywords:** Pubic symphysitis, Proteus mirabilis, Postoperative infection, MRI, Urological surgery, Pelvic pain

## Abstract

Abstract Infectious pubic symphysitis is a rare form of osteomyelitis, typically occurring after pelvic or urological surgery. We report the case of a 60-year-old man who developed acute pubic symphysitis following transurethral resection of the prostate (TURP) for benign prostatic hyperplasia. He presented with severe pelvic pain, fever, and gait difficulty. MRI revealed bone marrow edema of the pubic symphysis, and urine culture identified Proteus mirabilis. The patient was treated successfully with a 6 week antibiotic course, including 2 weeks of intravenous ceftriaxone followed by oral therapy, resulting in complete clinical recovery. This case underscores the importance of considering infectious pubic symphysitis in patients with persistent pelvic pain after urological procedures. Early diagnosis using MRI and targeted antibiotic therapy are key to preventing complications and ensuring favorable outcomes.

## Introduction

Infectious pubic symphysitis is a rare form of osteomyelitis involving the pubic symphysis, accounting for less than 1% of hematogenous osteomyelitis cases [1]. It was first described by Elliston in 1827 and is often associated with pelvic surgery, trauma, or systemic infections Urological procedures, such as radical prostatectomy and transurethral resection of the prostate, are recognized risk factors due to their proximity to the pubic symphysis and potential for urinary tract infections [2]. Diagnosis is challenging because symptoms are often nonspecific, including pelvic pain and fever, and requires a combination of clinical suspicion, laboratory tests, and imaging modalities like MRI, Treatment involves prolonged antibiotic therapy, and surgery is reserved for complicated cases .

## Case report

A 60-year-old man with benign prostatic hyperplasia refractory to medical therapy underwent transurethral resection of the prostate (TURP). A few days post-procedure, he presented to the emergency department with severe pelvic girdle pain, fever at 38 °C, and difficulty walking.

On examination, he was alert, hemodynamically and respiratory stable (BP 140/90 mmHg, HR 90 bpm, RR 18/min, normal oxygen saturation), but reported excruciating pelvic pain not relieved by first- and second-line analgesics. Physical exam revealed localized tenderness over the pubic symphysis without superficial abscess signs.

**Fig. 1:**
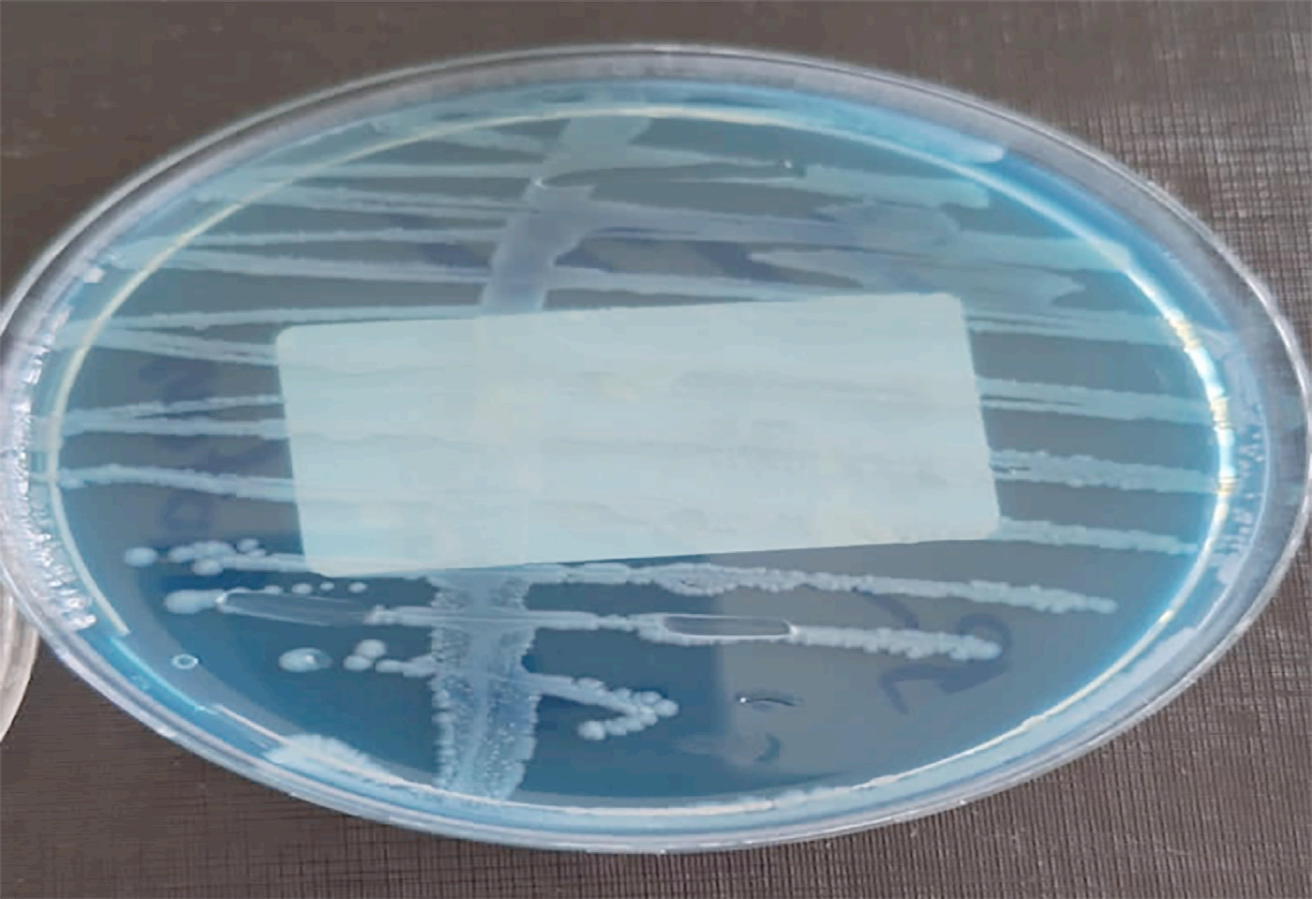
*Proteus mirabilis* culture on chromogenic agar showing characteristic colonies with a swarming appearance.

Urinalysis showed leukocyturia (150,000/mL) and bacteriuria (20,000/mL). Urine culture was positive for *Proteus mirabilis* sensitive to ceftriaxone (Fig. 1). Laboratory tests revealed leukocytosis (11,000/mm³) and elevated C-reactive protein (40 mg/L).

**Fig. 2:**
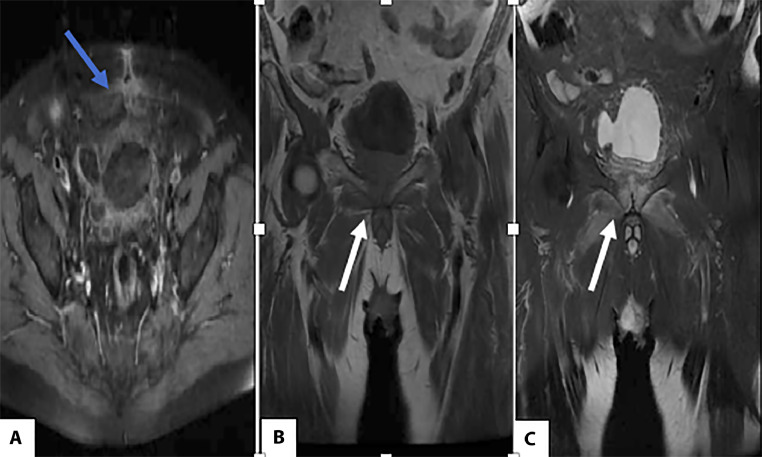
Axial section, post-contrast T1-weighted sequence (A). Infiltration of the pelvic region following urologic surgery. (B) and (C): Signal abnormality of the pubic symphysis, with low signal on T1 and high signal on T2, consistent with bone marrow edema.

Pelvic CT was unremarkable, but MRI showed a proton density hypersignal and T1 hyposignal, bilateral and asymmetric, centered on the subchondral bone of the pubic symphysis—consistent with infectious pubic symphysitis (Fig. 2).

A diagnosis of post-TURP infectious pubic symphysitis secondary to urinary tract infection with *Proteus mirabilis* was made.

The patient was treated with intravenous ceftriaxone 2 g/day by slow infusion. His clinical course was rapidly favorable with resolution of fever, marked pain improvement, and return to ambulation by day 5. Treatment was continued orally after 2 weeks of intravenous therapy, for a total of 6 weeks.

## Discussion

The first descriptions of infectious pubic symphysitis date back to Elliston in 1827 [1]. It accounts for less than 1% of all hematogenous osteomyelitis cases, and its true incidence in the general population is difficult to establish due to imprecise and inconsistent terminology in the literature [2]. Since then, multiple case reports have highlighted the diversity of etiologies and clinical presentations.

For instance, Allen et al. reported a case of pubic symphysitis following radical prostatectomy complicated by *Pseudomonas aeruginosa* infection [3], emphasizing the need for close postoperative monitoring. Kumar et al., in a 2018 review, described a series of 12 patients with varied etiologies including urological surgery, intravenous drug use, and obstetric infections, stressing the importance of prompt treatment to avoid complications such as abscess formation or septic dissemination [[4], [5]].

Pubic symphysitis often occurs in settings that favor local damage or infection of the symphysis. Known predisposing factors include uro-gynecological surgeries near the pubic region (radical prostatectomy via suprapubic or perineal approaches, transurethral resections, prostate biopsies, cystectomy, urinary incontinence surgeries, or inguinal hernia repairs) [6], pelvic malignancies complicated by tissue necrosis or digestive fistulas, obstetric maneuvers (delivery, abortion), intravenous drug use, and less commonly mechanical causes (athletes, pregnancy), infections (prostatitis, pyelonephritis), and rheumatologic diseases (rheumatoid arthritis, ankylosing spondylitis) [7,8].

When related to surgery, symphysitis is frequently associated with complications such as urinary tract infection, surgical site infection, or urethral/bladder perforation [9]. In our reported case, it was secondary to a urinary tract infection.

Biological markers are neither specific nor consistently present. Most patients show an inflammatory syndrome with elevated ESR and leukocytosis above 11,000/mm³.

Imaging starts with standard pelvic X-rays, which typically reveal changes after 2-4 weeks. Early radiographic signs include widening of the symphyseal joint space, blurred articular margins with erosions, and heterogeneous subchondral bone demineralization that may extend to the ischiopubic and iliopubic rami. Later stages show osteosclerosis and periosteal reactions, sometimes leading to synostosis.

CT scans, being more sensitive, better visualize soft tissue involvement, bone sequestra, abscesses, and complications and can guide biopsy.

MRI is particularly valuable for early detection of bone and muscle edema and inflammation, as well as joint effusions or abscesses [10], as demonstrated in our patient.

Causative pathogens may be identified through blood cultures or, in postpartum cases, from lochia. When cultures are negative, local sampling via trocar-guided biopsy or surgical approach is indicated.

Commonly isolated organisms include *Staphylococcus aureus* (especially in athletes with skin entry points), *Pseudomonas aeruginosa* (intravenous drug users or post-urological surgery), *Escherichia coli, Proteus mirabilis* (post-urological procedures), Group B Streptococcus and anaerobes (postpartum), and polymicrobial infections (especially in pelvic cancers) [7].

Treatment involves prolonged antibiotic therapy once the causative organism is identified. Most authors recommend an initial 2-week intravenous course tailored to the pathogen or clinical context, followed by oral therapy if clinical progress is favorable. Although no consensus exists on total treatment duration, a minimum of 6 weeks is generally accepted.

Surgical curettage may be necessary in cases of medical treatment failure, presence of sequestra, intraosseous foreign bodies, or abscesses. Symphysectomy has been proposed but is rarely required and may cause secondary pelvic instability [11].

Prognosis depends on whether the condition is acute or chronic. Without proper treatment, complications can include secondary septic localizations such as hip arthritis, adductor pyomyositis, or sacroiliitis [7].

Even with adequate antibiotics, persistent pubic pain may occur. Some symptoms resolve spontaneously over 2-3 years; others require additional anti-inflammatory treatment or corticosteroid injections.

It is important to note that radiological improvement often lags behind clinical recovery, with bone repair becoming visible only after 9-12 months.

## Conclusion

Although rare, infectious pubic symphysitis should be systematically considered in any patient presenting with prolonged or atypical pelvic pain after urological surgery, especially when associated with infectious or inflammatory signs. Diagnosis relies on appropriate imaging and microbiological identification. Prompt initiation of prolonged antibiotic therapy is essential to prevent serious complications and improve functional prognosis. This case highlights the importance of vigilant postoperative monitoring and a multidisciplinary approach involving urologists, radiologists, and infectious disease specialists to optimize patient outcomes.

## Ethical approval

No ethical approval is required for de-identified single case reports based on our institutional policies.

## Patient consent

Written informed consent was obtained from a legally authorized representative(s) for anonymized patient information to be published in this article.
